# Differential transcript and soluble factor patterns in macrophage/enterocyte-like monolayer co-cultures based on apical or basolateral LPS exposure

**DOI:** 10.3389/fimmu.2025.1527007

**Published:** 2025-02-20

**Authors:** Aurora Mazzei, Martina Cucchiara, Lorenzo Mortara, Elena Bossi, Roberta Schiavone, Tiziano Verri, Antonino Bruno, Amilcare Barca

**Affiliations:** ^1^ Applied Physiology Laboratory, Department of Biological and Environmental Sciences and Technologies, University of Salento, Lecce, Italy; ^2^ Laboratory of Innate Immunity, Unit of Molecular Pathology, Biochemistry and Immunology, Istituto di Ricovero e Cura a Carattere Scientifico (IRCCS) MultiMedica, Milan, Italy; ^3^ Immunology and General Pathology Laboratory, Department of Biotechnology and Life Sciences, University of Insubria, Varese, Italy; ^4^ Unit of Molecular Pathology, Biochemistry and Immunology, Istituto di Ricovero e Cura a Carattere Scientifico (IRCCS) MultiMedica, Milan, Italy; ^5^ Laboratory of Cellular and Molecular Physiology, Department of Biotechnology and Life Sciences, University of Insubria, Varese, Italy; ^6^ Applied Physiology Laboratory, Department of Experimental Medicine, University of Salento, Lecce, Italy

**Keywords:** Caco-2 monolayer, THP-1 macrophages, LPS, inflammatory pathways, IBD

## Abstract

**Background:**

The monolayer of intestinal epithelial cells (IECs) plays a crucial role in controlling intestinal homeostasis, also by its interaction with the immune system, via paracrine cytokine production, thus driving innate responses by tissue-resident immune cells. Here, using a co-culture model, we investigated the interactions between differentiated Caco-2 cells in monolayer and macrophages, by mimicking the cross-talk between enterocytes and immune cells during gastrointestinal (GI) tract inflammation.

**Methods:**

Caco-2 mature monolayers grown on Transwell membranes were challenged with apical or basolateral LPS. After stimulations, the enterocyte-like monolayers were transferred in co-culture with THP-1 derived macrophages. The functional impact of treatments was evaluated in terms of monolayer’s permeability, expression of mRNAs related to inflammation and immune responses and analysis of immune soluble factors present in the co-culture media.

**Results:**

LPS effectively affected monolayer’s permeability and induced a pro-inflammatory transcriptional program in Caco-2 monolayers. Remarkably, THP-1 derived macrophages differentially responded based on the diverse directional source of LPS, previously administered to the Caco-2 monolayers. Basolateral sensing of LPS, by Caco-2 monolayers, induced specific increase of several pro-inflammatory factors such as NF-kB1, IL-6 and IL-8, at transcript level, in macrophages, while apical sensing triggering targeted increase of IL-1β expression. Significantly, the analysis of immune factors secreted in the co-culture media suggested that paracrine interactions between enterocyte-like monolayers and macrophages are differently driven based on the basolateral vs. apical inflammation, previously triggered by LPS against the epithelial monolayer, and thus involving different immune gene networks.

**Conclusions:**

Taken together, our results suggest a framework of interactions between IECs and macrophages, depending upon the “polarized” inflammatory dysregulation.

## Graphical Abstract

Transcripts and soluble factors are expressed in macrophage/enterocyte-like monolayer co-cultures showing different patterns differentially driven based on the apical or basolateral LPS exposure suffered by the enterocyte monolayer only.

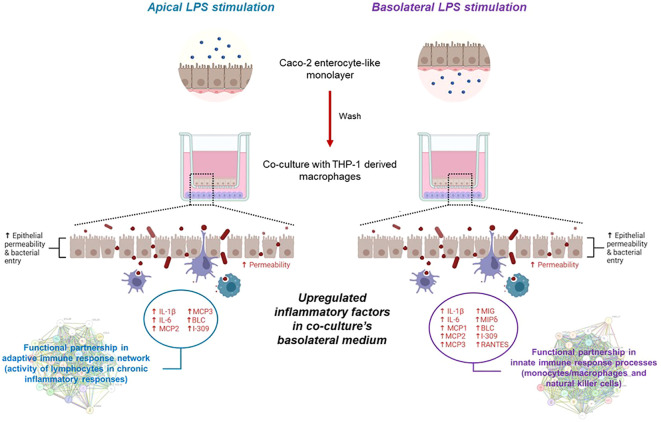

## 1 Introduction

Intestinal epithelial cells (IECs) make a continuous barrier that while driving transepithelial transport maintains immunomodulatory functions, acting as frontline sensors for microorganisms and integrating commensal bacteria-derived signals into anti-microbial and immuno-mediated and/or immunoregulatory responses (1). Any imbalance in this control can trigger aberrant dysregulations and inflammatory responses, as in the case of inflammatory bowel disease (IBD) (2). During initiation and propagation of intestinal inflammatory onsets, immune dysregulation is pivotal to the complex IBD pathogenesis (3). In such context, IECs influence the overall intestinal homeostasis and the cross-talk with immune cells, through reciprocal secretion of conditioning cytokines, affecting innate and adaptive responses primed by intestinal immune cells. In the healthy intestine, these conditioning factors help maintaining a state of hypo responsiveness towards commensal bacteria. Conversely, after sensing pathogenic invasion or damage, IECs can elaborate the secretion of pro-inflammatory chemokines, such as IL-8, that have an important role in alerting the immune system against microbial attack (4, 5).

Failure of intestinal immune regulation implies altered arrangement of the junctions between enterocytes, thus varying intestinal permeability (6). The function of intestinal barrier is primarily maintained by tight junctions (TJs), that seal the gap between neighboring cells. TJs are circumferential protein multiplexes, located at the apical and lateral compartments of IECs, thus controlling the structure and permeability of intestinal barrier (6). The expression and localization of these proteins in IECs are dynamically regulated by numerous intracellular and extracellular factors, including inflammatory cytokines, intestinal microorganisms and their metabolites (7).

When epithelial damage has occurred, TJs are compromised and without an effective barrier, gut luminal contents, pathogenic microbes and commensal microbiota are inappropriately translocated into the intestinal lamina propria, leading to inflammation (8). Thus, increased epithelial permeability is an established consequence of mucosal inflammation. Actually, in biopsies of IBD patients and in experimental models of colitis impaired structure and function of epithelial apical junctions have been observed (9, 10). Elimination of E-cadherin in mice enhanced cell death and loss of the intestinal mucosal architecture (11). Similarly, disruption of epithelial cell-cell adhesions and induction of mucosal inflammation has been reported in mice with intestinal epithelial KO of p120-catenin (an essential regulator of cadherin stability) (12), demonstrating that down-regulation of junction proteins’ expression represents an important mechanism of epithelial barrier disruption during inflammation.

The study of intestinal inflammation has made important progress, thanks to animal models. However, *in vitro* cell culture models are widely used in bioavailability and toxicological studies in both nutrition and pharmaceutical fields. Among them, the human derived Caco-2 cells represent an established system for investigating *in vitro* GI absorption, epithelial permeability and morpho-functional responses to physiological and pathophysiological stimuli (13, 14). Caco-2 cells are able to undergo spontaneous differentiation over time, starting few days after seeding and completing at 21 days of culture, gradually switching their morphological phenotype from the “tumor-like” to the “enterocyte-like” phenotype. This latter morpho-functionally recapitulates the absorptive monolayer of human small intestine, developing microvilli, TJs and expressing brush border associated enzymes (15, 16). To further improve simple cellular models, co-culture models have been developed for gut inflammation, by establishing the association of intestinal and immune cells, that represent the second most numerous cell type in the entire intestinal epithelial set. These models facilitate the study of cellular contact mechanisms and responses to soluble factors such as cytokines (17). Epithelial-immune co-cultures are optimally (and widely) employed to investigate the *in vitro* effects, on IECs, mediated by inflammatory molecular actors, such as IL-1β, TNF-α, IFN-γ and LPS. In Caco-2 cell monolayers, these stimuli have been evaluated with respect to the activation of intracellular cascades, leading to increased secretion of inflammatory cytokines that were found in association to the increase of the paracellular permeability, as assessed in terms of defects in TJ functioning or assembly (18–20).

Here, the characterization of a Caco-2/THP-1 derived macrophages co-culture model was performed, for mimicking the cross-talk between epithelial monolayer and innate immune cells, under LPS stimulation, focusing the analyses on the epithelial monolayer responses and on the immune cell responses induced by the challenged monolayer. To this aim, Caco-2 mature monolayers, i.e., at 21 days post-seeding (dps) grown in Transwell systems were challenged through the inflammatory/immunogen agent LPS at the apical or basolateral compartment and, after stimulations, transferred in co-culture with THP-1 derived macrophages. The functional impact of treatments was evaluated in terms of monolayer’s permeability, expression of genes related to inflammation and immune responses and analysis of immune soluble factors present in the co-culture media to evaluate the interactions between intestinal epithelial cells and immune cells during GI tract inflammation.

## 2 Materials and methods

### 2.1 Reagents and materials

All chemicals, reagents and kits were purchased at cell culture/molecular biology grade. Plasticware was invariably purchased sterilized, disposable and treated for cell culture. Fetal bovine serum (FBS), Dulbecco’s phosphate buffer saline (D-PBS), Eagle’s minimum essential medium (MEM), Roswell Park Memorial Institute (RPMI) 1640 medium, penicillin/streptomycin solutions, trypsin, L-glutamine and non-essential amino acids were purchased from Corning-Fisher Scientific (Rodano, MI, Italy). *E. coli*-derived lipopolysaccharide (LPS) and recombinant human interleukin 1-beta (IL-1β) were purchased from Thermo Fisher Scientific (Monza, Italy).

### 2.2 Cell line culture and maintenance

The Human epithelial Caco-2 cells (ATCC n. HTB-37™) were grown at 37°C, in a humidified atmosphere (5% CO2 in air), in MEM supplemented with 10% (v/v) FBS, 2 mM L-glutamine, 100 µg/ml penicillin-streptomycin and 1% (v/v) non-essential amino acid mix solution. The culture medium was replaced every second day (starting from the seeding day) and propagation occurred routinely every 4-5 days; then, after the third passage of propagation, for the experimental treatments, Caco-2 cells were used after continuous growth for 21 days post seeding (dps) to obtain a spontaneously differentiated intestinal epithelial monolayer (“enterocyte-like”) according to standard Caco-2 cell differentiation protocols (14, 15). THP-1 cells (ATCC n. TIB-202™) were maintained growing in suspension at 37°C in a humidified atmosphere (5% CO_2_ in air) in RPMI 1640 medium supplemented with 10% (v/v) FBS, 2 mM L-glutamine and 100 µg/ml penicillin-streptomycin-amphotericin B solution. The propagation occurred every 2-3 days; all the experiments were conducted between passage 3 and 10 of propagation. All cell lines used in this study were routinely screened for eventual mycoplasma contamination and only mycoplasma negative cells were used for subsequent experiments.

### 2.3 Caco-2/THP-1-derived macrophages co-cultures system and treatments

Caco-2 cells were seeded at 0.4 x 10^5^ cells/well on Transwell^®^ inserts (12 mm Transwell with 0.4 µm pore polyethylene terephthalate (PET) membrane insert, Corning-Mouser, Assago, MI, Italy) and maintained up to 21 dps until cells were fully differentiated. The culture medium was changed every 3-4 days. In the apical (A) side, cells were cultured in MEM, whereas the medium in the basolateral (BL) compartment was progressively changed to RPMI-based THP-1 medium.

**Figure 1:**
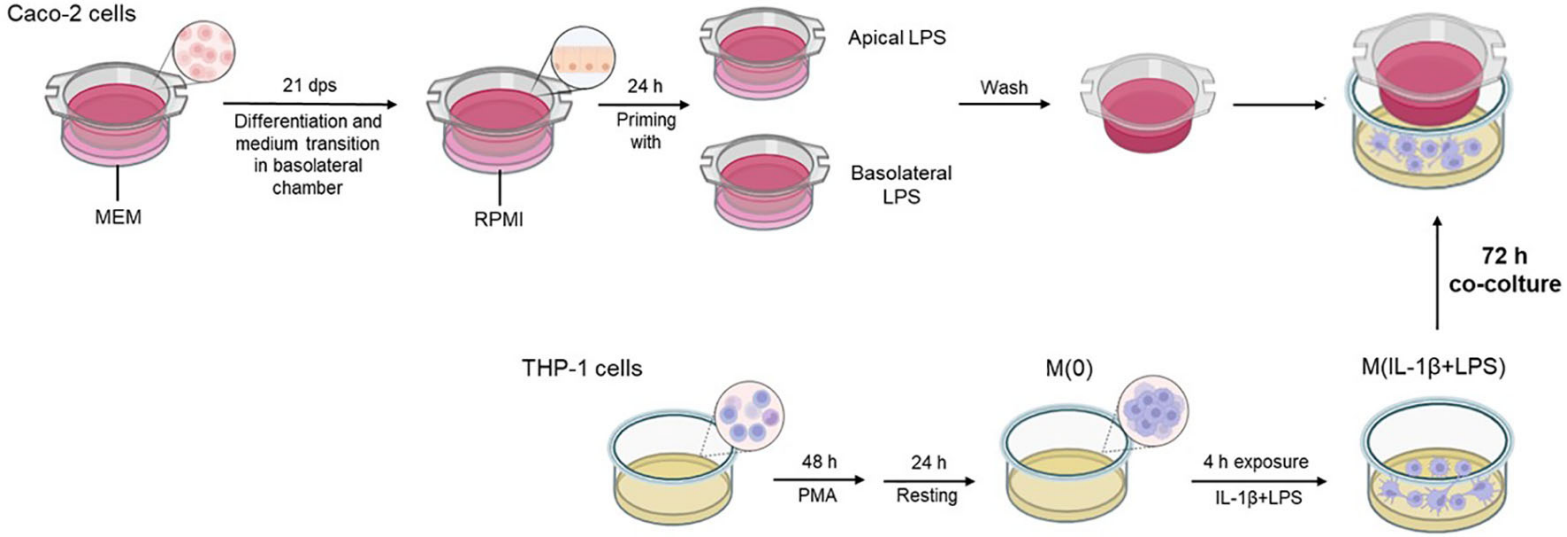
Graphical flow chart illustrating the co-culture system and related protocol. The differentiated Caco-2 monolayers are differentially primed with LPS in apical or basolateral compartments for 24 h before the start of the co-culture. At the end of the priming treatments, Caco-2 monolayers are washed for removal of the previous priming media and transferred onto multi-well plates containing the PMA-differentiated THP-1 cells, pre-exposed to IL-1β+LPS (4 h) before the initiation of co-culture. The co-culture was maintained for 72 h.

Twenty-one dps Caco-2 monolayers were differentially treated with 10 µg/mL LPS in apical or basolateral medium. After 24 h incubation, medium from A and BL compartments was removed and the Transwell inserts, containing primed Caco-2 monolayers, were washed twice with fresh medium before starting the co-culture with macrophages. In parallel, THP-1 monocytic cells were seeded into 12-well plates (4 x 10^5^ cells/well) and differentiated towards the macrophage phenotype by treatment with 50 nM PMA in RPMI medium for two days. The cells were then washed twice with fresh culture medium followed by 24 h rest in complete RPMI medium. Then, 4 h before starting co-culture with primed Caco-2 cell layers (from the previously described treatments), the macrophages were simultaneously pre-stimulated with IL-1β and LPS (10 ng/mL each). After 4 h pre-stimulation, inserts with primed Caco-2 were added into 12-well plates containing macrophages, and co-culture was maintained for 72 h (**Figure 1**). At the end of the experiment, Caco-2 cells from the inserts and macrophages were separately collected for downstream analyses.

### 2.4 Evaluation of cell monolayer integrity

The cell monolayer integrity was controlled by transepithelial electrical resistance (TEER) measurement performed with Millicell ERS-2 (Merck Millipore, USA) to check the barrier development of the Caco-2 monolayer (every 7 days of culture before the beginning of the experiments) as well as the barrier integrity throughout the co-culture with THP-1 derived macrophages (after 18, 24, 36, 48, 60 and 72 h). The electrode was sterilized in ethanol absolute (15 min) and neutralized in 0.15M NaCl and MEM. The results were adjusted for the blank and multiplied by the filter size (1.12 cm^2^), to express the final results as Ohm per cm^2^ (Ω*cm^2^).

### 2.5 Total RNA and protein extraction

Simultaneous RNA and protein extractions from cell cultures were performed using the All-Prep DNA/RNA/Protein mini kit (Qiagen) according to the manufacturer’s instructions. Briefly, cells grown in multi-well plates or Transwell inserts were washed twice with D-PBS and then lysed with the kit lysis buffer by scraping directly on the plate/insert surface. At the end of the RNA/protein extraction protocols, RNA aliquots were stored in RNase-free conditions at -80°C until use. The RNA concentration was determined with the NanoDrop ND-2000 Spectrophotometer (Nanodrop Technologies) and the λ_260_/λ_280_ ratios were calculated to evaluate RNA purity; all the RNA extractions were qualitatively tested by electrophoresis of RNA samples on 1% (w/v) agarose gels. Protein concentrations in extracts were calculated by the Bio-Rad Protein Assay Dye Reagent Concentrate according to the manufacture’s protocol.

### 2.6 Primer design and quantitative real PCR

The mRNA reference sequences of the investigated genes were collected from the GenBank database (https://www.ncbi.nlm.nih.gov/gene) and used to select oligonucleotide sequences as primer pairs for real-time PCR (qPCR) assays. By mRNA-to-genomic sequence alignment, gene-specific forward and reverse primers were designed on different exons (intron spanning) to avoid amplification of genomic DNA. The AmplifX software version 2.0.7 was used to test PCR size, GC content, end stability, self/cross-dimer formation and melting temperature for the selected primer pairs. Details of the gene-specific oligonucleotide sequences are reported in **Table 1**. Reverse transcription on the extracted RNAs was performed on 500-700 ng total RNA of each sample, using the iScript Select cDNA Synthesis kit (Bio-Rad, Segrate, MI, Italy) according to the manufacturer’s instructions, with random primers in the reaction mix. Before qPCR analysis, primer pairs were tested for efficiency, according to the amplification efficiency parameters for genes of interest and internal controls proposed by Schmittgen and Livak (21). qPCR assays were performed using the iTaq Universal SYBR Green Supermix (Bio-Rad) with a CFX96 Touch™ Real-Time PCR Detection System (Bio-Rad). In the qPCR analysis, gene expression relative quantification was performed by analyzing the threshold values (C_T_) with the comparative C_T_ method (also referred to as the 2^-ΔCT^ or 2^-ΔΔCT^ method), and qPCR data shown were the 2^-ΔCT^ values, which are considered as proportional to the amount of detected target mRNA. For each target gene and internal control (GAPDH, as housekeeping), ΔC_T_ values (ΔC_T_ = target gene C_T_ – housekeeping gene C_T_) were obtained from 2 different rounds of qPCR for each of three biological replicates. Statistical analysis was performed after the 2^-ΔCT^ transformation (21).

**Table 1:** Features of primer sequences for qPCR expression analysis.

GENE	RefSeq mRNA Acc. No.	Sense primer 5’-3’ (Tm)	Antisense primer 5’-3’ (Tm)	PCR size (bp)
*NFKB1b*	NM_003998.4	AATGCCTTCCGGCTGAGTC (59°C)	AGGCTGCCTGGATCACTTCA (60°C)	140
*IL1b*	NM_000576.3	CCTTCATCTTTGAAGAAGAACC (51°C)	GAGGTGGAGAGCTTTCAG (51°C)	158
*IL6*	NM_000600.5	GATGCTTCCAATCTGGATTC (51°C)	CAGGAACTGGATCAGGAC (51°C)	164
*MCP-1*	NM_002982.4	CCCCAGTCACCTGCTGTTAT (56°C)	TCCTGAACCCACTTCTGCTT (55°C)	166
*IL-8*	NM_000584.4	GTGCAGTTTTGCCAAGGAGT (56°C)	CTCTGCACCCAGTTTTCCTT (55°C)	196
*28S*	M27830.1	ACCCGAAAGATGGTGAACTA (52°C)	GCGAAAGACTAATCGAACCAT (53°C)	130
*GAPDH*	NM_002046.7	AAACCTGCCAAGTATGATGA (51°C)	TACTCCTTGGAGGCCATGT (54°C)	217

For each gene, the NCBI accession numbers of the mRNA reference sequence (RefSeq mRNA) used for primer design are reported. For each primer, the 5’-3’ nucleotide sequence and melting temperature (Tm) are reported. For each mRNA detection, the expected amplicon length is reported (PCR size) in base pairs (bp).

### 2.7 Analysis of protein products in media from co-cultures

As previously described (22, 23), conditioned media were characterized, in term of released soluble factor content, using the commercially available Human Cytokine Array C6 (RayBiotec, Peachtree Corners, GA, USA), according to the manufacturer instructions. Chemiluminescent signals (revealed as black dots) were captured by membrane exposure to Amersham Hyperfilm. Arrays were computer scanned using the Amersham Imager 680 Analyzer and optical density was determined using the ImageJ software.

### 2.8 *In silico* network analysis by STRING

Protein-Protein Interaction (PPI) Network Analysis was performed using the STRING online suite (STRING CONSORTIUM 2023^©^, https://string-db.org/, version 12.0, accessed on 27 September 2024) for PPI networks and functional enrichment analysis (24). Two PPI networks were constructed: 1) based on 6 protein products IL1B, IL6, MCP2 (aka CCL8), MCP3 (aka CCL7), BLC (aka CXCL13), I-309 (aka CCL1) (network stats: number of nodes 6; number of edges 13; average node degree 4.33; avg. local clustering coefficient 0.9; expected number of edges 2; PPI enrichment *p* value 8.39e^-08^); 2) based on 10 protein products IL1B, IL6, MCP1 (aka CCL2), MCP2 (aka CCL8), MCP3 (aka CCL7), BLC (aka CXCL13), I-309 (aka CCL1), MIG (aka CXCL9), MIP1 Delta (aka CCL15), RANTES (aka CCL5) (network stats: number of nodes 10; number of edges 41; average node degree 8.2; avg. local clustering coefficient 0.933; expected number of edges 5; PPI enrichment *p* value < 1.0e^-16^).

### 2.9 Statistical analysis

Unless otherwise specified, all results were shown as means ± standard deviation (SD). Data means derive from two independent assays, for each of three biological replicates. Statistical analysis was performed by one-way ANOVA, followed by Dunnett’s multiple comparison test, using GraphPad Prism 9.4.0. *p* value ≤ 0.05 was considered as statistically significant.

## 3 Results

### 3.1 Effects of LPS pre-treatments on epithelial permeability of Caco-2 monolayers in co-cultures

**Figure 2:**
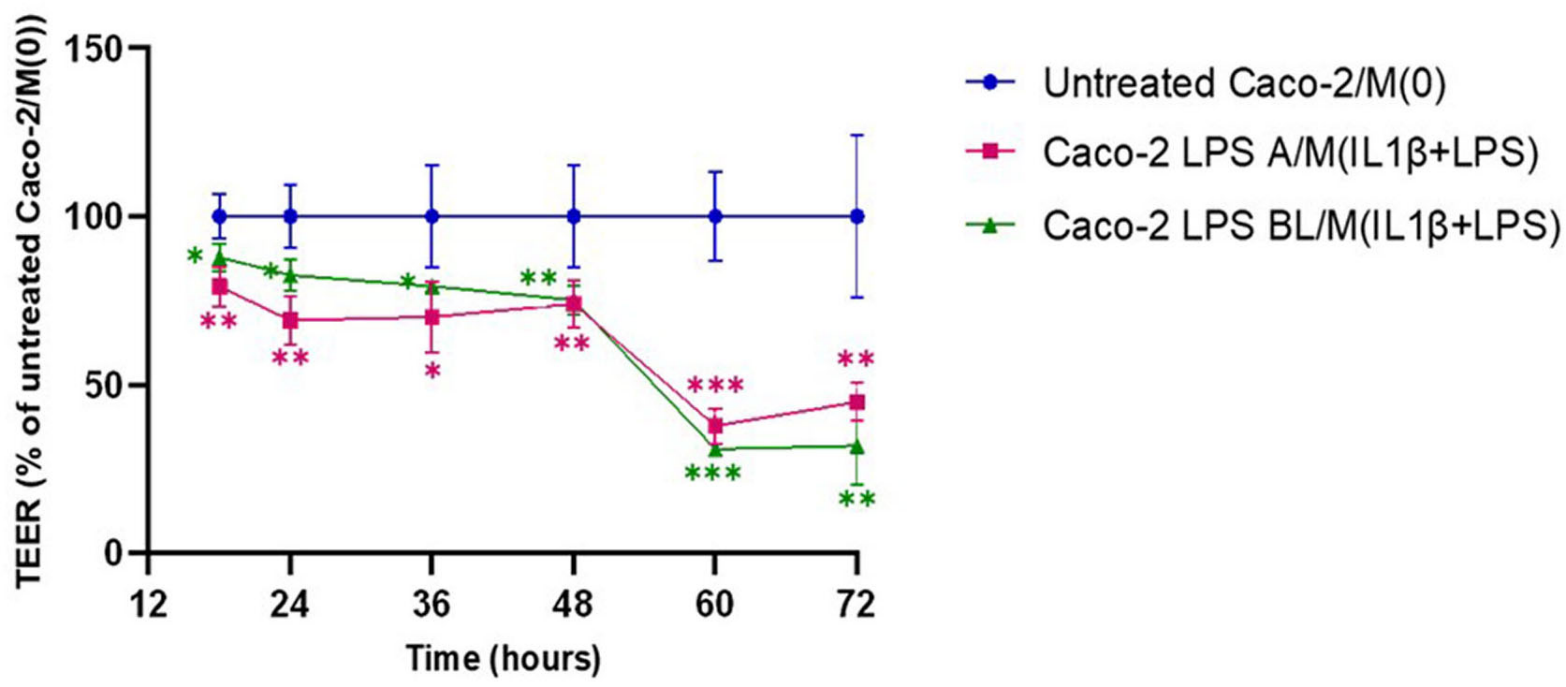
Monitoring of barrier integrity by TEER measurements in Caco-2 monolayers pre-exposed to apical/basolateral LPS over 72h co-culture with activated macrophages. Untreated Caco-2/M(0): untreated control co-culture; Caco-2 LPS A/M(IL-1β+LPS): Caco-2 cells primed with apical LPS before co-culture with THP-1 derived macrophages (primed with IL-1β+LPS); Caco-2 LPS BL/M(IL-1β+LPS): Caco-2 cells primed with basolateral LPS before co-culture with THP-1 derived macrophages (primed with IL-1β+LPS). Data are presented as mean ± SD of 3 independent biological replicates and then expressed as percent at each time point with respect to untreated control co-culture (100%). Statistical analysis: one-way ANOVA with Dunnett correction for multiple comparisons (*p < 0.05; **p < 0.01; ***p < 0.001).

To mimic proinflammatory stimulation on the intestinal barrier function *in vitro*, injury to a Caco-2 enterocyte-like monolayer was induced by LPS, before establishing a co-culture with macrophages. Epithelial barrier functional integrity was determined in differentiated Caco-2 monolayers, challenged by apical or basolateral LPS, for 24 h and, then co-cultured for 72 h with THP-1 derived macrophages [M(0)], stimulated by IL-1β/LPS. Effects of treatments on permeability of the Caco-2 epithelial monolayer were evaluated by TEER measurements. To initiate the co-culture, the Transwell filters with differentiated Caco-2 cells were transferred to well plates containing THP-1 derived M(0) macrophages, without additional manipulation of either cell lines. Then, TEER values were measured starting at 18 h and every 12 h, from 24 to 72 h co-culture. Overall, the analysis showed a TEER reduction in untreated Caco-2 monolayers co-cultured with M(0) macrophages, compared to Caco-2 monoculture alone (**Supplementary Material S1**, **Supplementary Figure 1**). Following 18 h co-culture, in Caco-2 monolayers previously treated with LPS both in apical (LPS A) and basolateral (LPS BL) compartments, a significant TEER decrease was detected, i.e., 79.23 ± 5.84% (*p* < 0.05) for LPS A and 87.85 ± 4.28% (*p* < 0.01) for LPS BL, compared to 100 ± 6.67% of untreated Caco-2 cells/M(0) control co-cultures (**Figure 2**). This reduction trend of TEER was constantly recorded during all time points, up to 48 h. As shown in **Figure 2**, starting from 60 h co-culture and up to the 72 h endpoint, a remarkably steep decrease in transepithelial resistance was recorded, indicating an LPS-induced Caco-2 barrier disruption, regardless the apical or basolateral LPS priming to Caco-2 monolayers (i.e., 44.88 ± 5.71% with LPS A and 31.98 ± 11.82% with LPS BL; *p* < 0.001) compared to the untreated co-culture (100 ± 24.29%).

### 3.2 Differential transcriptional modulation of inflammatory and immunoregulatory factors in co-cultured Caco-2 monolayers

The mRNA expression of inflammation-related factors (IL1B, interleukin-1 beta; IL6, interleukin-6; IL8, interleukin-8; MCP1, monocyte chemoattractant protein-1; NFkB1, nuclear factor kappa B subunit 1) was investigated, by qPCR, in apical/basolateral LPS-treated Caco-2 monolayers, after 72 h co-culture under the experimental conditions, as described in **Figure 1**.

**Figure 3:**
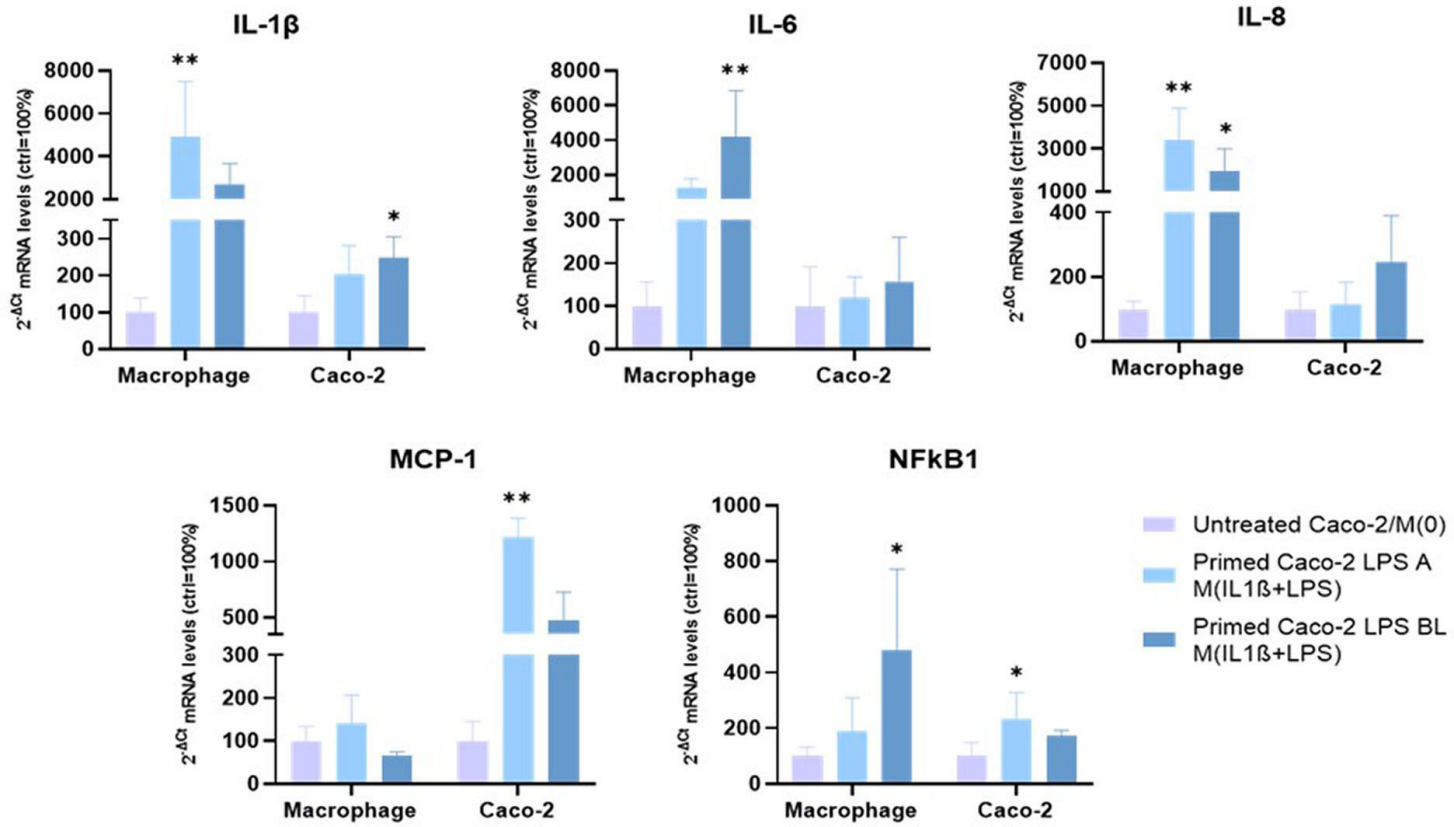
*mRNA expression analysis of inflammation-related gene, following 72 h co-culture.* Experimental condition: see colour code. Amounts of target mRNAs was calculated as 2^-ΔCt^ mean values obtained from 2 rounds of real-time PCR assays for each of 3 independent biological replicates and normalized with respect to the 28S rRNA gene for macrophages and GAPDH for Caco-2 cells. Then, values were expressed as percent with respect to the untreated Caco-2/M(0) co-culture mean value (100%). Statistical analysis by one-way ANOVA with Dunnett correction for multiple comparison (*p < 0.05; **p < 0.01).

As shown in **Figure 3**, IL1B mRNA levels were significantly up-regulated in Caco-2 monolayers, primed with basolateral LPS and then co-cultured with activated macrophages, compared to untreated Caco-2, co-cultured with M(0) macrophages (248.3 ± 57.2% with p<0.05, *vs.* 100 ± 45.6%). Conversely, MCP1 and NFκB1 mRNA levels were significantly up-regulated in Caco-2 monolayers primed with apical LPS and then co-cultured with activated macrophages compared to untreated Caco-2/M(0) macrophages (1215.6 ± 172.21% with *p* < 0.01 *vs.* 100 ± 45.7% for MCP1; 232.7 ± 94.9% with *p* < 0.05 *vs* 100 ± 48.5% for NFκB1). After 72 h coculture, IL6 and IL8 mRNA levels were not altered in Caco-2 monolayers primed with both apical and basolateral LPS stimulation.

### 3.3 Differential transcriptional modulation of inflammatory and immunoregulatory factors in co-cultured THP-1 derived macrophages

The same mRNA expression analyses were performed in the other co-culture cellular component, i.e., THP-1 derived macrophages (**Figure 3**). IL1B mRNA underwent strong up-regulation, when macrophages were co-cultured with LPS-primed Caco-2 monolayers. Up-regulation was statistically significant, following apical LPS priming (4905.7 ± 2586.9% with *p* < 0.01), compared to 100% of M(0) macrophages in untreated Caco-2/M(0) co-culture. IL6 and NFkB1 mRNAs levels were significantly increased in activated macrophages co-cultured with Caco-2 cells pre-exposed to basolateral LPS respect to M(0) macrophages (4217.5 ± 2632.9% with *p* < 0.01 for IL6, and 478.0 ± 293.0% with *p* < 0.05 for NFkB1, *vs.* 100% of respective controls). On the other hand, IL-8 expression underwent a strong, statistically significant up-regulation when macrophages were co-cultured with Caco-2 primed with both apical LPS and basolateral LPS (3420.5 ± 1451.7%, *p* < 0.01; 1982.9 ± 1011.4, *p* < 0.05, respectively) compared to 100% of M(0) in Caco-2/M(0) control co-culture. MCP1 mRNA levels showed no significant differences in both cases.

### 3.4 Analysis of the release of inflammatory and immunoregulatory soluble factors in apical and basolateral co-culture media

**Figure 4:**
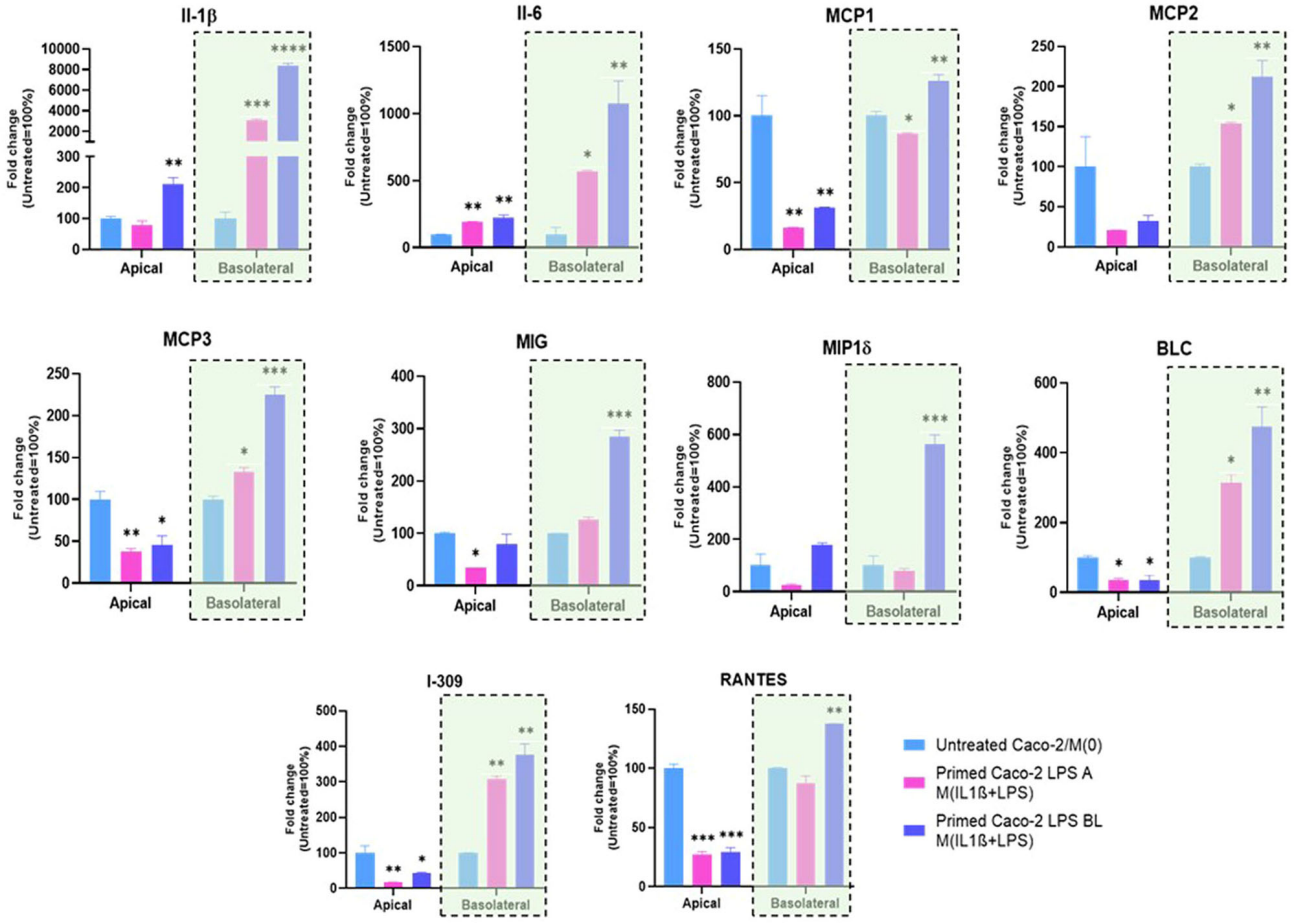
Expression of inflammation-related proteins released in conditioned media of the Caco-2/macrophage co-culture system. In histograms, Apical and Basolateral indicate cell culture media collected from the respective compartments after 72 h Caco-2/THP-1 derived macrophages co-culture. Primed Caco-2 LPS A M(IL-1β+LPS): culture condition with Caco-2 cells pre-treated by apical LPS exposure before co-culture; Primed Caco-2 LPS BL M(IL-1β+LPS): culture condition with Caco-2 cells pre-treated by basolateral LPS exposure before co-culture. Data from the basolateral media are highlighted (violet rectangles). Data are presented as mean ± SD of 3 independent biological replicates and then expressed as percent fold change with respect to the untreated Caco-2/M(0) co-culture mean value (100%). Statistical analysis by one-way ANOVA and Durrett correction for multiple comparisons (*p < 0.05; **p < 0.01; ***p < 0.001; ****p < 0.0001).

To further investigate the interaction between intestinal epithelial cells and macrophages, we collected the apical and basolateral conditioned media at the 72 h experimental endpoint of the co-culture system and analyzed them through a secretome profiler human cytokine array. The analysis of inflammatory soluble factors, released in the co-culture system, was performed by measuring levels of IL-1β, IL-6, MCP1, MCP2, MCP3, MIG, MP1δ, BLC, I-309, and RANTES protein products (**Figure 4**). The overall secretome analysis revealed their differential expression in apical *vs.* basolateral compartment of the co-culture. Specifically, in medium from the apical side we observed a prevailing downregulation of pro-inflammatory factors after co-culture, when Caco-2 were pre-stimulated with both apical and basolateral LPS, compared to the medium from untreated Caco-2/M(0) control co-culture (**Figure 4**), except for IL-1β and IL-6, that were found both up-regulated by LPS basolateral pre-stimulation. On the other hand, the secretion of soluble factors was found to be predominantly increased, in a statistically significant manner, in the medium from the basolateral side. More in detail, the secretion of pro-inflammatory factors in the basolateral side was more strongly and significantly induced, once Caco-2 monolayers were pre-stimulated with basolateral LPS than with apical, compared to the control Caco-2/M(0) unstimulated co-culture As shown in **Figure 4**, IL-1β, IL-6, MCP1, MCP2, MCP3, MIG, MIP1δ, BLC, I-309, and RANTES were all found to be up-regulated in the basolateral medium with statistical significance, after enterocyte/macrophage co-culture upon basolateral pre-stimulation of the Caco-2 monolayer; conversely, only the IL-1β, IL-6, MCP2, MCP3, BLC, and I-309 protein levels (i.e., 6 out of 10) were found statistically increased in the basolateral medium, after enterocyte/macrophage co-culture upon apical pre-stimulation of the monolayer.

### 3.5 Network analysis of up-regulated protein products in the basolateral medium

Based on the two groups of differentially upregulated proinflammatory factors as assessed in the basolateral medium after 72 h Caco-2/macrophage co-culture depending on the apical vs. basolateral LPS pretreatment applied to the Caco-2 monolayer, a network analysis was performed by the STRING online tool to infer protein-protein interaction networks and functional enrichment analysis.

**Figure 5:**
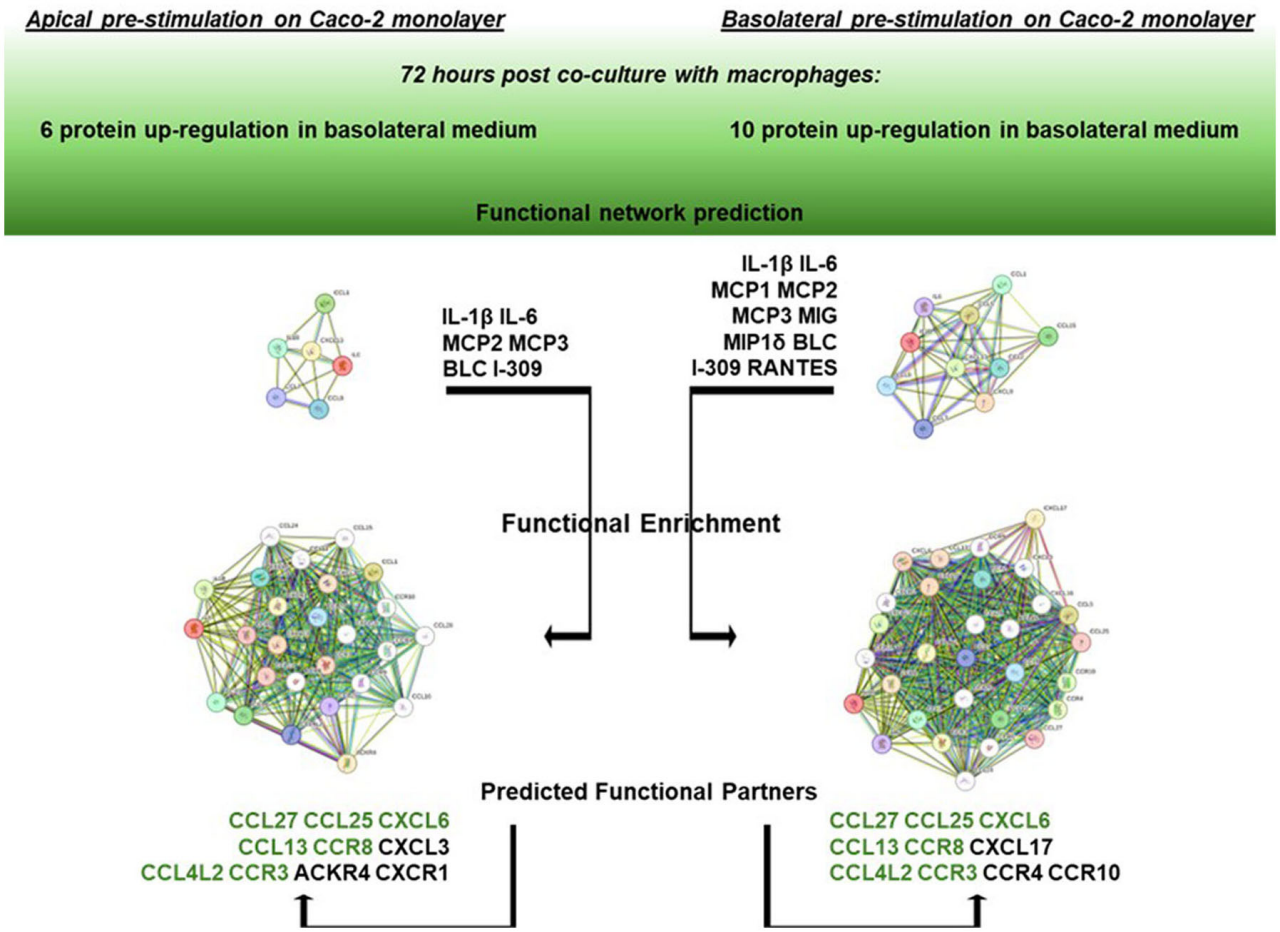
Network enrichment analysis and prediction of functional partners by STRING. On the left: STRING functional enrichment of the 6 gene product network (IL-1β, Il-6, MCP2, MCP3, BLC, and I-309) with the best 10 predicted functional partners. On the right: STRING functional enrichment of the 10 gene product network (IL-1β, Il-6, MCP1, MCP2, MCP3, MIG, MIP1δ, BLC, I-309, and RANTES). Detailed descriptions and scores are reported in **Supplementary Material S1**. Common predicted functional partners are depicted in green.

As shown in **Figure 5**, starting from the core network of 6 gene products found upregulated in the co-culture basolateral medium when the Caco-2 monolayer was apically pre-exposed to LPS, the functional enrichment analysis of the interactors showed the best 10 predicted functional partners being CCL27, CCL25, CXCL6, CCL13, CCR8, CXCL3, CCL4L2, CCR3, ACKR4, and CXCR1 (for STRING scores and functional details, see **Supplementary Material S1**). On the other hand, starting the analysis from the core network of 10 gene products found upregulated when the Caco-2 monolayer was pre-exposed to LPS from the basolateral side, the functional enrichment analysis showed the best 10 predicted interactors being CCL27, CCL25, CXCL6, CCL13, CCR8, CXCL17, CCL4L2, CCR3, CCR4, and CCR10. Apart from the seven common elements (CCL27, CCL25, CXCL6, CCL13, CCR8, CCL4L2), the analysis starting from 6 gene products (corresponding to apical LPS pre-stimulation of the enterocytes) indicated 3 specific functional partners, i.e., CXCL3, ACKR4 and CXCR1. The STRING gene ontology data refer their interactivity to the biological processes of a) migration/positive regulation of lymphocyte chemotaxis, and b) chronic inflammatory response (see **Supplementary Material S1** for full description and scores). In parallel, the analysis starting from 10 gene products (corresponding to basolateral LPS pre-stimulation of the enterocytes) indicated 3 different specific functional partners, i.e., CXCL17, CCR4 and CCR10: in this case, the STRING gene ontology data indicate relations with the biological processes of a) (positive) regulation of natural killer cell chemotaxis, and b) positive regulation of cell-cell adhesion mediated by integrin (see **Supplementary Material S1** for full description and scores).

## 4 Discussions

The interaction between epithelial cells and macrophages is a dynamic and highly regulated process essential for both the initiation and resolution of inflammation in the GI tract. Epithelial cells act as sentinels, sensing inflammatory signals and recruiting macrophages, while macrophages, in turn, respond by producing both pro-inflammatory and tissue-repairing mediators, based on the surrounding cytokine/chemokine and growth factors milieu. Dysregulation of this interaction can lead to chronic inflammation and pathological conditions such as IBD. Proper modulation of this crosstalk is crucial for maintaining mucosal homeostasis and preventing disease progression that, if not contained, can result in an exacerbated chronic inflammatory state, also potentially resulting in cancer of the GI tract (2).

In this work, we report results deriving from a co-culture model of Caco-2 differentiated monolayers and THP-1 derived macrophages, for mimicking the cross-talk between epithelial and immune cells under LPS stimulation as inflammation causative agent. The enterocyte-like monolayers were previously challenged with apical or basolateral LPS and only subsequently co-cultured with activated macrophages.

The LPS basolateral priming of enterocyte-like cells in monolayer, in advance of the co-culture, mimics the pathophysiological conditions in which bacterial antigens have already translocated across the intestinal epithelial cell barrier and subsequent activation of immune cells occurs from the blood compartment; conversely, LPS apical priming of enterocyte-like cells mimics the sensing of external antigens normally occurring in the microbiota/epithelium interactions. In our study, LPS effectively affected monolayer’s permeability regardless the apical or basolateral exposure, but, remarkably, macrophages differentially responded in terms of expression of products of inflammatory genes, based on the different directional source of LPS previously administered to Caco-2 monolayers. In fact, in terms of transcriptional expression, basolateral sensing of LPS in Caco-2 cells induces a more specific up-regulation of IL-6, IL-8 and NFκB1 inflammatory mRNAs in M(IL-1β+LPS) macrophages, while apical involves IL-1β and IL-8. On the other hand, both apical or basolateral LPS do not show specific transcriptional activations in Caco-2 monolayers (see **Figure 3**).

Afterwards, the detection of soluble factors by secretome arrays, in apical/basolateral co-culture media, was performed. In the basolateral compartment, data revealed a remarkable increase of pro-inflammatory soluble factors when the Caco-2 monolayer was challenged by basolateral LPS. In particular, IL-1β, Il-6, MCP1, MCP2, MCP3, MIG, MIP1δ, BLC, I-309, and RANTES were analysed and all ten found to be up-regulated together in the basolateral medium, after enterocyte/macrophage co-culture upon basolateral pre-stimulation of the Caco-2 monolayer. These up-regulated factors are involved in several aspects of inflammation and immune activation (interleukins, monocyte chemoattractant proteins, pro-angiogenic chemokines, macrophage inflammatory proteins, lymphocyte chemoattractant, chemotactic cytokines for natural killer cells). Importantly, the secretion of all these pro-inflammatory factors in the basolateral side was more strongly and significantly induced, once Caco-2 monolayers were exposed to basolateral LPS than to apical LPS, compared to Caco-2/M(0) unstimulated co-culture (see **Figure 4**). Moreover, levels of only 6 out of 10 of those factors (i.e., IL-1β, Il-6, MCP2, MCP3, BLC, and I-309) were found increased in the basolateral medium after enterocyte/macrophage co-culture, upon apical pre-stimulation of the Caco-2 monolayer. It should be noted, finally, that no peculiar immune regulatory pattern of the genes under examination was identified in the apical medium, neither following apical stimulation of the monolayer, nor basolateral stimulation.

In this work, an *in silico* network analysis session was conducted to better clarify the biological significance of the two different groups of genes differentially upregulated depending on the directionality of the LPS stimulus and the possible differential downstream pathways. Remarkably, by functional enrichment analysis of the core network of the 6 upregulated gene products in the co-culture basolateral medium after Caco-2 monolayer’s apical pre-exposure to LPS, a different set of functional partners was identified compared to the core network of 10 upregulated genes after Caco-2 monolayer’s basolateral pre-exposure to LPS. In detail, taking into account the interacting proteins with the best ten functional partner scores, the functional enrichment of the 6 gene core network showed the involvement of three factors (CXCL3, ACKR4, CXCR1; see **Figure 5**) whose gene ontology analysis refers to biological processes related to lymphocyte migration/chemotaxis in the framework of chronic inflammatory responses. Differently, the functional enrichment starting from the 10 gene core network elicits the recruitment in network of factors referring to processes related to activity of natural killer cells (CXCL17, CCR4, CCR10, in **Figure 5**). These diverse functional involvements give rise to a crucially interesting interpretation of the differential responses observed in terms of factors released in the basolateral medium: if the inflammatory stimulus (LPS) previously administered to the intestinal monolayer is apical, this leads, following co-culture with macrophages, to an upregulation of soluble factors (6 gene products) potentially related to an adaptive immune response network (lymphocytes in chronic inflammatory responses); on the contrary, if the monolayer is previously exposed to a basolateral inflammatory stimulus, this leads, following cross-talk in co-culture with macrophages, to an upregulation of soluble factors (10 gene products) interacting in innate immune response processes (monocytes/macrophages and natural killer cells). In this sense, the concerted upregulation of the two specifically different sets of gene products could represent a marker situation of the two different activations implemented by the enterocyte/macrophage system in co-culture, depending on the different signals that the enterocyte monolayer can communicate, depending on whether it “upstream” perceives a luminal proinflammatory signal (apical) or whether it perceives it systemically (basolateral). Nevertheless, as TEER measurements demonstrate, the two situations do not exert different trends in affecting epithelial barrier permeability; that is, the different (apical *vs*. basolateral) sensing of LPS does not elicit different enterocyte barrier integrity, but it is differentially interpreted once the barrier initiates its cross-talk with activated macrophages.

## 5 Conclusions

The pathophysiological effects of LPS on the Caco-2/macrophage co-culture give hints of inflammatory features including those of the IBD, e.g., epithelial barrier dysfunction, recruitment of monocytes and increased function of pro-inflammatory pathways. These characteristics were exhibited by the co-culture model described here, in which macrophages respond to stimuli previously perceived by the enterocyte-like cells. By discriminating the apical *vs*. basolateral source of the inflammatory agent, noteworthy, this model system might contribute to clarify different responses or signaling of the affected enterocytes in different phases of the IBD related inflammation framework.

The limitation of this study might be represented by the fact that other inflammatory agents should be compared to LPS to find out converging or diverging evidences. In fact, future directions will include investigating stimuli such as interferons or inflammatory metabolites derived from diet. Besides this, the evidences assessed by our model might be exploited to characterize the time-dependent features of enterocyte/macrophage cross-talk in inflammatory context *in vitro*, to be easily relocated in a parallel model *in vivo*.

Comprehensively, our work also allows generating and establishing a valid *in vitro* tool to investigate the bi-directional enterocyte-macrophage interactions and crosstalk that could be employed to study cellular and molecular alterations occurring in the pathogenesis and progression of inflammatory-driven pathology of the GI tract, including cancer.

## Data Availability

The raw data supporting the conclusions of this article will be made available by the authors, without undue reservation.

## References

[1] Peterson LW Artis D. Intestinal epithelial cells: regulators of barrier function and immune homeostasis. Nat Rev Immunol. (2014) 14:141–53. doi: 10.1038/nri3608 24566914

[2] Maloy KJ Powrie F. Intestinal homeostasis and its breakdown in inflammatory bowel disease. Nature. (2011) 474:298–306. doi: 10.1038/nature10208 21677746

[3] Guan Q. A comprehensive review and update on the pathogenesis of inflammatory bowel disease. J Immunol Res. (2019) 2019:7247238. doi: 10.1155/2019/7247238 31886308 PMC6914932

[4] Moldoveanu AC Diculescu M Braticevici CF. Cytokines in inflammatory bowel disease. Rom J Intern Med. (2015) 53:118–27. doi: 10.1515/rjim-2015-0016 26402980

[5] Friedrich M Pohin M Powrie F. Cytokine networks in the pathophysiology of inflammatory bowel disease. Immunity. (2019) 50:992–1006. doi: 10.1016/j.immuni.2019.03.017 30995511

[6] Kuo WT Odenwald MA Turner JR Zuo L. Tight junction proteins occludin and ZO-1 as regulators of epithelial proliferation and survival. Ann N Y Acad Sci. (2022) 1514:21–33. doi: 10.1111/nyas.v1514.1 35580994 PMC9427709

[7] Chelakkot C Ghim J Ryu SH. Mechanisms regulating intestinal barrier integrity and its pathological implications. Exp Mol Med. (2018) 50:1–9. doi: 10.1038/s12276-018-0126-x PMC6095905 30115904

[8] Serek P Oleksy-Wawrzyniak M. The effect of bacterial infections, probiotics and zonulin on intestinal barrier integrity. Int J Mol Sci. (2021) 22:11359. doi: 10.3390/ijms222111359 34768787 PMC8583036

[9] Das P Goswami P Das TK Nag T Sreenivas V Ahuja V. Comparative tight junction protein expressions in colonic Crohn's disease, ulcerative colitis, and tuberculosis: a new perspective. Virchows Arch. (2012) 460:261–70. doi: 10.1007/s00428-012-1195-1 22297703

[10] Kuo WT Zuo L Odenwald MA Madha S Singh G Gurniak CB. The tight junction protein ZO-1 is dispensable for barrier function but critical for effective mucosal repair. Gastroenterology. (2021) 161:1924–39. doi: 10.1053/j.gastro.2021.08.047 PMC8605999 34478742

[11] Schneider MR Dahlhoff M Horst D Hirschi B Trülzsch K Müller-Höcker J. A key role for E-cadherin in intestinal homeostasis and Paneth cell maturation. PLoS One. (2010) 5:e14325. doi: 10.1371/journal.pone.0014325 21179475 PMC3001873

[12] Smalley-Freed WG Efimov A Burnett PE Short SP Davis MA Gumucio DL. p120-catenin is essential for maintenance of barrier function and intestinal homeostasis in mice. J Clin Invest. (2010) 120:1824–35. doi: 10.1172/JCI41414 PMC2877948 20484816

[13] Jochems PGM Garssen J van Keulen AM Masereeuw R Jeurink PV. Evaluating human intestinal cell lines for studying dietary protein absorption. Nutrients. (2018) 10:322. doi: 10.3390/nu10030322 29518965 PMC5872740

[14] Hidalgo IJ Raub TJ Borchardt RT. Characterization of the human colon carcinoma cell line (Caco-2) as a model system for intestinal epithelial permeability. Gastroenterology. (1989) 96:736–49. doi: 10.1016/S0016-5085(89)80072-1 2914637

[15] Sambuy Y De Angelis I Ranaldi G Scarino ML Stammati A Zucco F. The Caco-2 cell line as a model of the intestinal barrier: influence of cell and culture-related factors on Caco-2 cell functional characteristics. Cell Biol Toxicol. (2005) 21:1–26. doi: 10.1007/s10565-005-0085-6 15868485

[16] Natoli M Leoni BD D'Agnano I D'Onofrio M Brandi R Arisi I. Cell growing density affects the structural and functional properties of Caco-2 differentiated monolayer. J Cell Physiol. (2011) 226(6):1–43. doi: 10.1002/jcp.22487 20945374

[17] Ponce-de-León-Rodríguez MDC Guyot JP Laurent-Babot C. Intestinal *in vitro* cell culture models and their potential to study the effect of food components on intestinal inflammation. Crit Rev Food Sci Nutr. (2019) 59:3648–66. doi: 10.1080/10408398.2018.1506734 30277794

[18] Stephens M von der Weid PY. Lipopolysaccharides modulate intestinal epithelial permeability and inflammation in a species-specific manner. Gut Microbes. (2020) 11:421–32. doi: 10.1080/19490976.2019.1629235 PMC7524286 31203717

[19] Van De Walle J Hendrickx A Romier B Larondelle Y Schneider YJ. Inflammatory parameters in Caco-2 cells: effect of stimuli nature, concentration, combination and cell differentiation. Toxicol In Vitro. (2010) 24:1441–9. doi: 10.1016/j.tiv.2010.04.002 20406675

[20] Kämpfer AAM Urbán P Gioria S Kanase N Stone V Kinsner-Ovaskainen A. Development of an *in vitro* co-culture model to mimic the human intestine in healthy and diseased state. Toxicol In Vitro. (2017) 45:31–43. doi: 10.1016/j.tiv.2017.08.011 28807632 PMC5744654

[21] Schmittgen TD Livak KJ. Analyzing real-time PCR data by the comparative C_T_ method. Nat Protoc. (2008) 3:1101–8. doi: 10.1038/nprot.2008.73 18546601

[22] Gallazzi M Baci D Mortara L Bosi A Buono G Naselli A. Prostate cancer peripheral blood NK cells show enhanced CD9, CD49a, CXCR4, CXCL8, MMP-9 production and secrete monocyte-recruiting and polarizing factors. Front Immunol. (2021) 11:586126. doi: 10.3389/fimmu.2020.586126 33569050 PMC7868409

[23] Barone L Palano MT Gallazzi M Cucchiara M Rossi F Borgese M. Adipose mesenchymal stem cell-derived soluble factors, produced under hypoxic condition, efficiently support *in vivo* angiogenesis. Cell Death Discovery. (2023) 9:174. doi: 10.1038/s41420-023-01464-4 37221171 PMC10205717

[24] Szklarczyk D Kirsch R Koutrouli M Nastou K Mehryary F Hachilif R. The STRING database in 2023: protein-protein association networks and functional enrichment analyses for any sequenced genome of interest. Nucleic Acids Res. (2023) 51:D638–46. doi: 10.1093/nar/gkac1000 PMC9825434 36370105

