# Supplementary material for: Differential transcript and soluble factor patterns in macrophage/enterocyte-like monolayer co-cultures based on apical or basolateral LPS exposure
Source: Front Immunol. 2025 Feb 20;16:1527007. doi: 10.3389/fimmu.2025.1527007 (PMC11882427; doi:10.3389/fimmu.2025.1527007)
Supplement: Supplementary file 1 [file DataSheet1.docx]

Supplementary Material

Supplementary Materials S1

1. **Details and scores from STRING analysis. 10 gene core network (Basolateral pre-stimulation).**

Network stats post enrichment: number of nodes 30; number of edges 383; average node degree 25.5; avg. local clustering coefficient 0.909; expected number of edges 34; PPI enrichment p-value < 1.0e-^16^.

| **Predicted Functional Partners by STRING (best 10 scoring)** | | | |  |  |  |  |  |  |  |  |  |  |
| --- | --- | --- | --- | --- | --- | --- | --- | --- | --- | --- | --- | --- | --- |
|  |  | CCL27 | *C-C motif chemokine 27; Chemotactic factor that attracts skin-associated memory T- lymphocytes. May play a role in mediating homing of lymphocytes to cutaneous sites. Binds to CCR10.* |  |  |  |  |  |  |  |  | 0.999 |  |
|  |  | CCL25 | *C-C motif chemokine 25; Potentially involved in T-cell development. Recombinant protein shows chemotactic activity on thymocytes, macrophages, THP-1 cells, and dendritics cells but is inactive on peripheral blood lymphocytes and neutrophils. Binds to CCR9. Isoform 2 is an antagonist of isoform 1. Binds to atypical chemokine receptor ACKR4 and mediates the recruitment of beta-arrestin (ARRB1/2) to ACKR4; Belongs to the intercrine beta (chemokine CC) family.* |  |  |  |  |  |  |  |  | 0.998 |  |
|  |  | CXCL6 | *Small-inducible cytokine B6, N-processed variant 1; Chemotactic for neutrophil granulocytes. Signals through binding and activation of its receptors (CXCR1 and CXCR2). In addition to its chemotactic and angiogenic properties, it has strong antibacterial activity against Gram-positive and Gram-negative bacteria (90-fold-higher when compared to CXCL5 and CXCL7).* |  |  |  |  |  |  |  |  | 0.999 |  |
|  |  | CCL13 | *C-C motif chemokine 13, medium chain; Chemotactic factor that attracts monocytes, lymphocytes, basophils and eosinophils, but not neutrophils. Signals through CCR2B and CCR3 receptors. Plays a role in the accumulation of leukocytes at both sides of allergic and non-allergic inflammation. May be involved in the recruitment of monocytes into the arterial wall during the disease process of atherosclerosis. May play a role in the monocyte attraction in tissues chronically exposed to exogenous pathogens; Belongs to the intercrine beta (chemokine CC) family.* |  |  |  |  |  |  |  |  | 0.999 |  |
|  |  | CCR8 | *C-C chemokine receptor type 8; Receptor for the chemokine CCL1/SCYA1/I-309. May regulate monocyte chemotaxis and thymic cell line apoptosis. Alternative coreceptor with CD4 for HIV-1 infection.* |  |  |  |  |  |  |  |  | 0.999 |  |
|  |  | CXCL17 | *C-X-C motif chemokine 17; Chemokine that acts as chemoattractant for monocytes, macrophages and dendritic cells. Plays a role in angiogenesis and possibly in the development of tumors. Acts as an anti-inflammatory in the stomach. May play a role in the innate defense against infections. Activates the C-X-C chemokine receptor GPR35 to induce a rapid and transient rise in the level of intracellular calcium ions. Belongs to the intercrine alpha (chemokine CxC) family.* |  |  |  |  |  |  |  |  | 0.997 |  |
|  |  | CCL4L2 | *C-C motif chemokine ligand 4 like 2.* |  |  |  |  |  |  |  |  | 0.999 |  |
|  |  | CCR3 | *C-C chemokine receptor type 3; Receptor for C-C type chemokine. Binds and responds to a variety of chemokines, including CCL11, CCL26, CCL7, CCL13, RANTES(CCL5) and CCL15. Subsequently transduces a signal by increasing the intracellular calcium ions level. In addition acts as a possible functional receptor for NARS1.* |  |  |  |  |  |  |  |  | 0.999 |  |
|  |  | CCR4 | *C-C chemokine receptor type 4; High affinity receptor for the C-C type chemokines CCL17/TARC, CCL22/MDC and CKLF isoform 1/CKLF1. The activity of this receptor is mediated by G(i) proteins which activate a phosphatidylinositol-calcium second messenger system. Can function as a chemoattractant homing receptor on circulating memory lymphocytes and as a coreceptor for some primary HIV-2 isolates. In the CNS, could mediate hippocampal-neuron survival.* |  |  |  |  |  |  |  |  | 0.977 |  |
|  |  | CCR10 | *C-C chemokine receptor type 10; Receptor for chemokines SCYA27 and SCYA28. Subsequently transduces a signal by increasing the intracellular calcium ions level and stimulates chemotaxis in a pre-B cell line; Belongs to the G-protein coupled receptor 1 family.* |  |  |  |  |  |  |  |  | 0.910 |  |

**Functional enrichments in network (best 10 scoring)**

**Biological Process (Gene Ontology)**

| **GO TERM - DESCRIPTION** | ***count in network***  ***strength***  ***false discovery rate*** |
| --- | --- |
| **GO:0048245**  **Eosinophil chemotaxis** | **10 of**[**17**](https://string-db.org/cgi/network?network_term_id=GO:0048245&input_query_species=9606)  **2.59**  **4.83e-20** |
| **GO:1903237**  **Negative regulation of leukocyte tethering or rolling** | **2 of**[**4**](https://string-db.org/cgi/network?network_term_id=GO:1903237&input_query_species=9606)  **2.52**  **0.0032** |
| **GO:2000501**  **Regulation of natural killer cell chemotaxis** | **3 of 8**  **2.39**  **6.67e-05** |
| **GO:2000503**  **Positive regulation of natural killer cell chemotaxis** | **2 of 6**  **2.34**  **0.0057** |
| **GO:0033634**  **Positive regulation of cell-cell adhesion mediated by integrin** | **2 of**[**6**](https://string-db.org/cgi/network?network_term_id=GO:0033634&input_query_species=9606)  **2.34**  **0.0057** |
| **GO:0002548**  **Monocyte chemotaxis** | **14 of 43**  **2.33**  **4.40e-26** |
| **GO:0048247**  **Lymphocyte chemotaxis** | **15 of 51**  **2.29**  **1.41e-27** |
| **GO:0070098**  **Chemokine-mediated signaling pathway** | **24 of 82**  **2.28**  **9.19e-47** |
| **GO:0010573**  **Vascular endothelial growth factor production** | **2 of 7**  **2.27**  **0.0072** |
| **GO:0030593**  **Neutrophil chemotaxis** | **19 of 80**  **2.19**  **1.91e-34** |

1. **Details and scores from STRING analysis. 6 gene core network (Apical pre-stimulation)**

Network stats post enrichment: number of nodes 26; number of edges 287; average node degree 22.1; avg. local clustering coefficient 0.907; expected number of edges 28; PPI enrichment p-value < 1.0e-^16^.

| **Predicted Functional Partners by STRING (best 10 scoring)** | | | |  |  |  |  |  |  |  |  |  |  |
| --- | --- | --- | --- | --- | --- | --- | --- | --- | --- | --- | --- | --- | --- |
|  |  | CXCL6 | *Small-inducible cytokine B6, N-processed variant 1; Chemotactic for neutrophil granulocytes. Signals through binding and activation of its receptors (CXCR1 and CXCR2). In addition to its chemotactic and angiogenic properties, it has strong antibacterial activity against Gram-positive and Gram-negative bacteria (90-fold-higher when compared to CXCL5 and CXCL7).* |  |  |  |  |  |  |  |  | 0.997 |  |
|  |  | CCL27 | *C-C motif chemokine 27; Chemotactic factor that attracts skin-associated memory T- lymphocytes. May play a role in mediating homing of lymphocytes to cutaneous sites. Binds to CCR10.* |  |  |  |  |  |  |  |  | 0.913 |  |
|  |  | CCL4L2 | *C-C motif chemokine ligand 4 like 2.* |  |  |  |  |  |  |  |  | 0.996 |  |
|  |  | CCL25 | *C-C motif chemokine 25; Potentially involved in T-cell development. Recombinant protein shows chemotactic activity on thymocytes, macrophages, THP-1 cells, and dendritics cells but is inactive on peripheral blood lymphocytes and neutrophils. Binds to CCR9. Isoform 2 is an antagonist of isoform 1. Binds to atypical chemokine receptor ACKR4 and mediates the recruitment of beta-arrestin (ARRB1/2) to ACKR4; Belongs to the intercrine beta (chemokine CC) family.* |  |  |  |  |  |  |  |  | 0.874 |  |
|  |  | CCR8 | *C-C chemokine receptor type 8; Receptor for the chemokine CCL1/SCYA1/I-309. May regulate monocyte chemotaxis and thymic cell line apoptosis. Alternative coreceptor with CD4 for HIV-1 infection.* |  |  |  |  |  |  |  |  | 0.999 |  |
|  |  | CCL13 | *C-C motif chemokine 13, medium chain; Chemotactic factor that attracts monocytes, lymphocytes, basophils and eosinophils, but not neutrophils. Signals through CCR2B and CCR3 receptors. Plays a role in the accumulation of leukocytes at both sides of allergic and non-allergic inflammation. May be involved in the recruitment of monocytes into the arterial wall during the disease process of atherosclerosis. May play a role in the monocyte attraction in tissues chronically exposed to exogenous pathogens; Belongs to the intercrine beta (chemokine CC) family.* |  |  |  |  |  |  |  |  | 0.998 |  |
|  |  | CCR3 | *C-C chemokine receptor type 3; Receptor for C-C type chemokine. Binds and responds to a variety of chemokines, including CCL11, CCL26, CCL7, CCL13, RANTES(CCL5) and CCL15. Subsequently transduces a signal by increasing the intracellular calcium ions level. In addition acts as a possible functional receptor for NARS1.* |  |  |  |  |  |  |  |  | 0.999 |  |
|  |  | CXCL3 | *C-X-C motif chemokine 3; Ligand for CXCR2 (By similarity). Has chemotactic activity for neutrophils. May play a role in inflammation and exert its effects on endothelial cells in an autocrine fashion. In vitro, the processed form GRO-gamma(5-73) shows a fivefold higher chemotactic activity for neutrophilic granulocytes.* |  |  |  |  |  |  |  |  | 0.860 |  |
|  |  | ACKR4 | *Atypical chemokine receptor 4; Atypical chemokine receptor that controls chemokine levels and localization via high-affinity chemokine binding that is uncoupled from classic ligand-driven signal transduction cascades, resulting instead in chemokine sequestration, degradation, or transcytosis. Also known as interceptor (internalizing receptor) or chemokine-scavenging receptor or chemokine decoy receptor. Acts as a receptor for chemokines CCL2, CCL8, CCL13, CCL19, CCL21 and CCL25. Chemokine-binding does not activate G-protein-mediated signal transduction but instead induces beta-arrestin [...]* |  |  |  |  |  |  |  |  | 0.992 |  |
|  |  | CXCR1 | *C-X-C chemokine receptor type 1; Receptor to interleukin-8, which is a powerful neutrophils chemotactic factor. Binding of IL-8 to the receptor causes activation of neutrophils. This response is mediated via a G-protein that activate a phosphatidylinositol-calcium second messenger system. This receptor binds to IL-8 with a high affinity and to MGSA (GRO) with a low affinity.* |  |  |  |  |  |  |  |  | 0.987 |  |

**Functional enrichments in your network (best 10 scoring)**

**Biological Process (Gene Ontology)**

| **GO TERM - DESCRIPTION** | ***count in network***  ***strength***  ***false discovery rate*** |
| --- | --- |
| **GO:1903237**  **Negative regulation of leukocyte tethering or rolling** | **2 of 4**  **2.58**  **0.0031** |
| **GO:0048245**  **Eosinophil chemotaxis** | **7 of 17**  **2.49**  **3.06e-13** |
| **GO:0010573**  **Vascular endothelial growth factor production** | **2 of 7**  **2.34**  **0.0067** |
| **GO:0070098**  **Chemokine-mediated signaling pathway** | **21 of 82**  **2.29**  **6.79e-41** |
| **GO:0002548**  **Monocyte chemotaxis** | **11 of 43**  **2.29**  **9.15e-20** |
| **GO:0048247**  **Lymphocyte chemotaxis** | **12 of 51**  **2.25**  **2.17e-21** |
| **GO:0030593**  **Neutrophil chemotaxis** | **15 of 80**  **2.15**  **4.22e-26** |
| **GO:0072676**  **Lymphocyte migration** | **13 of 80**  **2.09**  **1.53e-21** |
| **GO:0002544**  **Chronic inflammatory response** | **2 of 13**  **2.07**  **0.0186** |
| **GO:0140131**  **Positive regulation of lymphocyte chemotaxis** | **3 of 22**  **2.01**  **0.00065** |

# Supplementary Figure 1


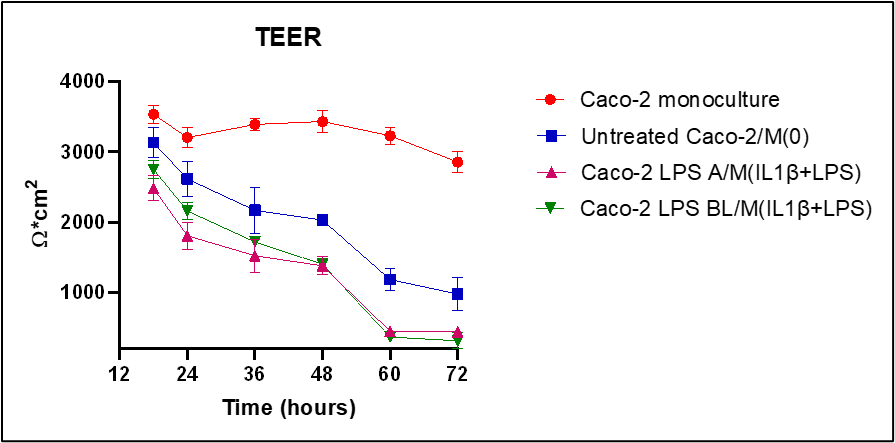


*Monitoring of barrier integrity by TEER measurements in Caco-2 monolayers pre-exposed to apical/basolateral LPS over 72h co-culture with activated macrophages*. Caco-2 monoculture: untreated control Caco-2 monoculture grown on Transwell membranes up to 22 dps (days post seeding). **Untreated Caco-2/M(0)**: untreated control co-culture; **Caco-2 LPS A/M(IL-1β+LPS)**: Caco-2 cells primed with apical LPS before co-culture with THP-1 derived macrophages (primed with IL-1β+LPS); **Caco-2 LPS BL/M(IL-1β+LPS)**: Caco-2 cells primed with basolateral LPS before co-culture with THP-1 derived macrophages (primed with IL-1β+LPS). Data are presented as mean ± SD of 3 independent biological replicates and then expressed as percent at each time point with respect to untreated control co-culture (100%). Statistical analysis: one-way ANOVA with Dunnett correction for multiple comparisons (**p* < 0.05; ***p* < 0.01; ****p* < 0.001).
